# Association of Serum Phosphorus Variability with Coronary Artery Calcification among Hemodialysis Patients

**DOI:** 10.1371/journal.pone.0093360

**Published:** 2014-04-18

**Authors:** Mengjing Wang, Haiming Li, Li You, Xiaoling Yu, Min Zhang, Ruijiang Zhu, Chuanming Hao, Zhijie Zhang, Jing Chen

**Affiliations:** 1 Division of Nephrology, Huashan Hospital, Shanghai Medical College, Fudan University, Shanghai, China; 2 Division of Radiology, Huashan Hospital, Shanghai Medical College, Fudan University, Shanghai, China; 3 Department of Epidemiology and Biostatistics, School of Public Health, Fudan University, Shanghai, China; 4 Biomedical statistical Center, Fudan University, Shanghai, China; Brigham and Women's Hospital, Harvard Medical School, United States of America

## Abstract

Coronary artery calcification (CAC) is associated with increased mortality in patients on maintenance hemodialysis (MHD), but the pathogenesis of this condition is not well understood. We evaluated the relationship of CAC score (CACs) and variability in serum phosphorus in MHD patients. Seventy-seven adults on MHD at Huashan Hospital (Shanghai) were enrolled in July, 2010. CAC of all the patients were measured by computed tomography and CACs was calculated by the Agatston method at the entry of enrollment. Patients were divided into three categories according to their CACs (0∼10, 11∼400, and >400). Blood chemistry was recorded every 3 months from January 2008 to July 2010. Phosphorus variation was defined by the standard deviation (SD) or coefficient of variation (CV) and it was calculated from the past records. The ordinal multivariate logistic regression analysis was used to analyze the predictors of CAC. The mean patient age (± SD) was 61.7 years (±11.3) and 51% of patients were men. The mean CACs was 609.6 (±1062.9), the median CACs was 168.5, and 78% of patients had CACs more than 0. Multivariate analysis indicated that female gender (OR = 0.20, 95% CI = 0.07–0.55), age (OR = 2.31, 95% CI = 1.32–4.04), serum fibroblast growth factor 23 (OR = 2.25, 95% CI = 1.31–3.85), SD-phosphorus calculated from the most recent 6 measurements (OR = 2.12; 95% CI = 1.23–3.63), and CV-phosphorus calculated from the most recent 6 measurements (OR = 1.90, 95% CI = 1.16–3.11) were significantly and independently associated with CACs. These associations persisted for phosphorus variation calculated from past 7, 8, 9, 10, and 11 follow-up values. Variability of serum phosphorus may contribute significantly to CAC and keeping serum phosphorus stable may decrease coronary calcification and associated morbidity and mortality in MHD patients.

## Introduction

Coronary artery calcification (CAC) is common in patients on maintenance hemodialysis (MHD) therapy [1]–[3], and such patients have increased risk for cardiovascular disease (CVD) and all-cause mortality [4]–[9]. Previous research indicated that dialysis patients had a 10- to 20-fold increased risk for death from CVD relative to age- and gender-matched members of the general population [10]. The mechanisms of CAC are not well understood, but advanced age, male sex, hypertension, dyslipidemia, chronic inflammatory state [1]–[3], [11], [12], dialysis duration [13], oxidative stress [11], bone-related proteins [14], [15], and mineral disturbances [2], [16] are associated with increased risk of CAC. However, some studies have refuted these reported associations [17], [18].

There is much controversy regarding the mechanism of CAC. Block *et al.* first reported a positive association of hyperphosphatemia and mortality in HD patients [19], and this led to subsequent studies of the association between CAC and phosphorus metabolism in uremic patients, but the results of these studies have been contradictory. In particular, Raggi *et al.* reported that the extent of coronary calcification was greater in MHD patients with higher serum concentrations of phosphorus [2] and Jung *et al.* reported that elevated serum phosphorus was associated with rapid progression of CAC in HD patients [20]. However, other studies reported no association of serum phosphorus and CAC in HD populations [1], [11], [15], [21], [22]. Thus, rigorous prospective clinical studies and outcome studies are needed to definitively establish the relationship of elevated serum phosphorus and CAC.

The level of serum phosphorus varies throughout the day under normal physiological conditions [23], but (in the absence of advanced chronic kidney disease [CKD]) is maintained within the range of 2.5 to 4.5 mg/dL (0.8–1.4 mmol/L) by a variety of mechanisms including gastrointestinal absorption, urinary excretion, bone loss and uptake, and transport between the intracellular and extracellular spaces [24]. The level of serum phosphorus has greater daily variation in HD patients due to increased gut absorption from high daily protein intake, high levels of active vitamin D, bone disorders, and decreased urinary excretion [25]. Thus, the serum phosphorus levels of HD patients fluctuate much more than in healthy individuals [26]. However, few studies have examined the relationship between the extent of variation in serum phosphorus and CAC.

The purpose of this study was to investigate the association of serum phosphorus variability with CAC in MHD patients.

## Materials and Methods

### Patients

This was a retrospective study of 77 consecutive Chinese HD patients in one hemodialysis center (Huashan Hospital, Fudan University, Shanghai, PR China) from January 2008 to July 2010. All patients were over 18 years-old, on HD more than 15 months, and were followed up every 3 months with biochemical and immunological testing on the same day. Patients were excluded if they had severe malnutrition, hepatic insufficiency, active infection, active malignancy, heart failure, prior history of coronary artery revascularization or myocardial infarction, vasculitis, or diabetes mellitus or hypertension that was poorly controlled. The Ethics Committee on Human Research at Huashan Hospital, Fudan University approved this study and all patients provided written informed consent. All patients were undergoing HD (4 h per session, 3 times per week) and were treated with bicarbonate dialysis fluid and low-flux dialysers made of polysulfone (surface area: 1.2 m^2^, Diacap α, B. Braun., Melsungen, Germany).

### Data collection and evaluation of variability in serum phosphorus, calcium and parathyroid hormone

Data on demographics and dialysis-specific and clinical characteristics were collected at the time of enrollment (July 2010). Two daily urine collections were pooled for the creatinine and urea clearance calculations. The GFR was estimated as the mean of creatinine and urea clearance adjusted for body surface area (ml/min per 1.73 m^2^) [25]. Biochemical parameters were also recorded at this time, including: serum hemoglobin, ferritin, transferrin saturation, carbon dioxide combining power (CO_2_CP), C-reactive protein (CRP), albumin, serum creatinine, blood urea nitrogen, total, high-density and low-density lipoprotein cholesterol (HDL and LDL), and triglycerides, phosphorus, calcium, parathyroid hormone (PTH), fibroblast growth factor 23 (FGF23), and 1,25(OH)_2_D_3_.

Variability was defined as the standard deviation (SD) or coefficient of variation (SD/mean) [27]. So the past testing results (every 3 months from January 2008 to July 2010, 11 times in total) of serum phosphorus, calcium, and PTH levels were also obtained. For each parameter, we calculated six CVs, six SDs, and six means based on the past 6, 7, 8, 9, 10, and 11 values.

### Imaging procedure

All patients were scanned using a 256-detector-row Brilliance iCT scanner (Philips Healthcare, Cleveland, OH) in July 2010. The entire heart was covered in a single breath-hold (20–30 sec). Slices of 3.0-mm thickness were acquired with 150 mA of tube current at 120 kV. Quantification was performed by a single trained reader who was blinded to the clinical data, using software for calcium scoring (Heartbeat-CS, EBW, Philips Medical Systems, Best, The Netherlands). This software can detect calcified lesions with a density of at least 130 Hounsfield units (HU) over a minimal area of 0.5 mm^2^. Patients were assigned calcification scores based on the number, area, and peak HU of the calcific lesions, as described by Agatston *et al* [28]. (1: 110–199 HU; 2: 200–299 HU; 3: 300–399 HU; 4: >400 HU). Data obtained during the diastolic phase of the heart cycle were used for image reconstruction. The total score was calculated by summing the calcification scores of the left main, left anterior descending, left circumflex, and right coronary arteries.

### Laboratory testing

The patients in the morning shift fasted routinely after 10 pm before the day of laboratory tests. However the patients in the afternoon shift had their lunch after the blood samples were collected. Blood samples were drawn direct from the AV-fistula at 8 am or 1 pm (at the beginning of dialysis) on the day of the mid-week HD session and biochemical parameters were assessed by standard techniques. The formula used to calculate corrected calcium was described in the K/DOQI guidelines [29]: CorrCa = Measured serum Ca+[4.0 - measured serum albumin (g/dL)]×0.8. The single-pool Kt/V_urea_ delivered by HD (sp-dKt/V_urea_) was estimated by the second-generation Daugirdas formula [30]. The normalized protein nitrogen appearance (nPNA) was calculated as described by Termorshuizen *et al.* and normalized to standard body weight (total body water/0.58) [31]. Total-body water was determined from Watson's formula [32]. The serum levels of intact PTH (Santa Cruz, CA, USA), intact FGF23 (Immutopics, San Clemente, CA, USA), and 1,25(OH)_2_D_3_ (Immunodiagnostic Systems, Boldon, UK) were measured by ELISA according to the manufacturers' protocols. The detection of intact FGF23 guarantees that we measure the biologically active form of human FGF23. FGF23 fragments will not be measured [33].

### Statistical analysis

The mean, SD, median, and interquartile range (IQR) or number and percentage were used to characterize variables of the study objects. Ordinal univariate logistic regression was first used to analyze the predictors of CAC, based on CACs. Explanatory variables included two types of data: *(i)* clinical characteristics and biochemical parameters obtained at the time of enrollment (age, sex, smoking, diabetes, dialysis vintage, body mass index [BMI], nPNA, systolic BP, diastolic BP, blood flow, Kt/V, medications, serum hemoglobin, ferritin, transferrin saturation, CO_2_CP, CPR, albumin, serum creatinine, blood urea nitrogen, phosphorus, calcium, PTH, FGF23, 1,25(OH)_2_D_3_, total, HDL, LDL, cholesterol, and triglycerides); and *(ii)* variations and means of serum phosphorus, calcium, and PTH calculated from previous records. The explanatory variables were not normally distributed, so the BOX-COX transformation was applied before modeling. Predictors with *p*-values of 0.2 or less were included in the ordinal multivariate logistic regression analysis. Backward selection was used to determine the significant variables in the best fitted model, and a *p*-value less than 0.05 was considered statistically significant. Six models were developed using the same explanatory variables described above except for variations and means of serum phosphorus, calcium, and PTH. The variations and means of six models were calculated from previous 6, 7, 8, 9, 10, and 11 values, respectively. All statistical analysis was performed with SAS version 9.3 (SPSS, Inc., Chicago, IL, USA).

## Results

### Coronary artery calcification and traditional risk factors

**Figure 1:**
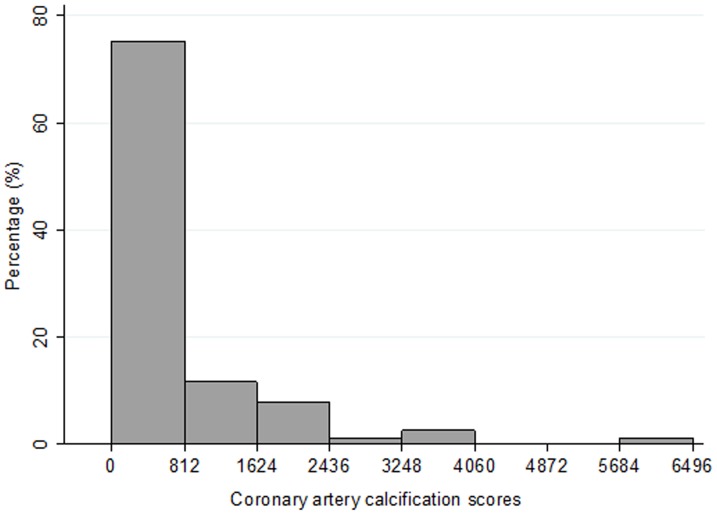
Distribution of coronary artery calcification scores (CACs) in MHD patients. Seventeen patients had no evidence of coronary calcification (CACs = 0) and 60 patients had CACs from 0.5 to 6493.2. Seventy five percent of the CACs were between 0 and 811.6, the mean CACs was 609.6±1062.9, the median CACs was 168.5, and the interquartile range was 2.5 to 788.2.

Of the whole patients that met the inclusion criteria, 5 patients were excluded for active infection, prior history of coronary artery revascularization and myocardial infarction. Seventy-seven patients were actually enrolled. The mean (±SD) age of the 77 patients was 61.7 (±11.3) years and 51% of patients were men. 82% of female patients were in menopausal or postmenopausal and they did not receive estrogen therapy. The causes of renal failure were diabetes mellitus (n = 11), hypertension (n = 10), glomerulonephritis (n = 45), and other conditions (n = 11). The average time on HD therapy was 5.9 years (±4.4) and the mean Kt/V was 1.47 (±0.21). Figure 1 shows the distribution of CACs for the 77 patients. Seventeen patients had no evidence of CAC and the other 60 patients had CACs from 0.5 to 6493.2. The mean CACs was 609.6 (±1062.9), the median was 168.5, and the IQR was 2.5 to 788.2.

We classified these patients into three coronary calcification groups using the modified categorization proposed by Rumberger *et al.* [34]: mild (CACs = 0 to 10, low risk for CVD), moderate (CACs = 11 to 400, moderate risk for CVD), and severe (CACs>400, severe risk for CVD). Table 1 shows the baseline characteristics of the study subjects based on CAC category. Male gender (*p*<0.001) and advanced age (*p* = 0.01) were significantly associated with higher CACs, but the other variables (dialysis vintage, smoking, diabetes, GFR, systolic and diastolic blood pressure, blood flow rate, Kt/V, and medications) had no significant associations with CACs.

**Table 1:** Baseline characteristics of MHD patients by CAC score tertile.

	CAC score			
	<10 (n = 23)	11–400 (n = 26)	>400 (n = 28)	*P*
Male (n %)	6 (26.1)	14 (53.8)	19 (67.8)	<0.001**
Age (years)	56.8±12.5	61.8±11.4	65.5±8.7	0.01**
Dialysis vintage (months)	69.5±63.9	69.5±48.8	74.6±46.7	0.7
Smokers (n)	2	6	9	0.05*
Diabetes (n %)	1 (4.3)	5 (19.2)	5 (17.8)	0.3
GFR (ml/min per 1.73 m^2^)	1.04±1.83	0.86±2.76	0.29±0.96	0.2
Systolic BP (mmHg)	117.0±19.1	117.1±28.6	119.3±22.5	0.8
Diastolic BP (mmHg)	71.9±10.8	67.7±12.7	72.4±11.4	0.8
Blood flow rate (mL/min)	226.2±20.9	231.4±30.3	235.9±20.3	0.2
Kt/V urea-dialysis	1.49±0.25	1.46±0.22	1.46±0.17	0.7
Dosage of CaCO_3_ (g/d)	2.7±2.7	2.9±2.8	2.9±2.9	0.8
Dosage of calcitriol (ug/w)	2.17±4.33	2.42±5.96	2.03±3.69	0.9

Values are expressed as mean ± SD or number (percentage).

MHD, maintenance hemodialysis; CAC, coronary artery calcification; BP, blood pressure.

Data compared by univariate ordinal logistic regressions;

*P<0.2,

**P<0.05.

Table 2 shows the laboratory measurements of patients in these three groups. The results indicate significant relationships between higher CRP (*p* = 0.04), lower serum albumin (*p* = 0.02), and lower serum HDL (*p* = 0.03) with higher CACs. Other measures of nutritional status (BMI and nPNA), iron parameters (serum ferritin and transferrin saturation), lipid metabolism (total cholesterol, triglycerides, and LDL), mineral metabolism (serum phosphorus, calcium, PTH, FGF23, 1,25(OH)_2_D_3_), serum creatinine, blood urea nitrogen did not differ significantly among the 3 groups at enrollment.

**Table 2:** Laboratory measures of MHD patients by CAC score tertile at study onset.

	CAC score			
	<10 (n = 23)	11–400 (n = 26)	>400 (n = 28)	*P*
Hb (g/dL)	10.8±1.2	10.7±1.8	11.2±1.7	0.5
Ferritin (ng/ml)	322.2±188.0	394.4±193.5	408.9±180.4	0.1*
Transferrin saturation (%)	38.7±11.3	40.2±14.6	40.3±15.2	0.7
CO_2_CP (mmol/L)	22.6±2.1	21.4±2.1	21.9±3.2	0.4
CRP (mg/L)	3.6±1.6	6.0±5.6	10.1±13.6	0.04**
BMI (kg/m^2^)	20.7±3.2	21.1±2.8	20.9±2.3	0.8
nPNA (g/kg per day)	1.06±0.20	1.12±0.15	1.07±0.18	0.9
Scr (umol/L)	864±173	945±236	929±188	0.3
BUN (mmol/L)	23.5±5.0	24.8±3.8	25.1±4.3	0.2
Alb (g/dL)	3.91±0.28	3.73±0.25	3.72±0.29	0.02**
Total cholesterol (mg/dL)	175.6±41.0	160.1±36.0	161.6±36.0	0.2
Triglycerides (mg/dL)	187.8±116.9	154.1±86.8	194.0±118.7	0.7
LDL-cholesterol (mg/dL)	87.8±34.0	81.6±26.3	82.0±24.8	0.5
HDL-cholesterol (mg/dL)	39.8±11.2	36.7±11.6	32.1±8.1	0.03**
Phosphorus (mg/dL)	5.18±1.38	5.23±1.58	5.31±1.32	0.8
Calcium (mg/dL)	9.21±0.91	9.22±0.87	9.52±0.82	0.2
PTH (pg/mL)	160.4±91.7	236.3±244.1	282.7±258.3	0.09*
FGF23 (pg/mL)	250.8±334.5	413.5±352.3	429.5±373.9	0.1*
1,25(OH)_2_VitD_3_ (pg/mL)	3.22±3.93	4.23±4.22	3.23±4.23	0.9

Values expressed as mean ± SD.

MHD, maintenance hemodialysis; CAC, coronary artery calcification; Hb, hemoglobin; CO_2_CP, carbon dioxide combining power; CRP, C-reactive protein; BMI, body mass index; nPNA, normalized protein nitrogen appearance; Alb, albumin; Scr, Serum creatinine; BUN, blood urea nitrogen; LDL, low-density lipoprotein; HDL, high-density lipoprotein; PTH, parathyroid hormone; FGF23, fibroblast growth factor 23.

Data compared by univariate ordinal logistic regressions;

*P<0.2,

**P<0.05.

**Figure 2:**
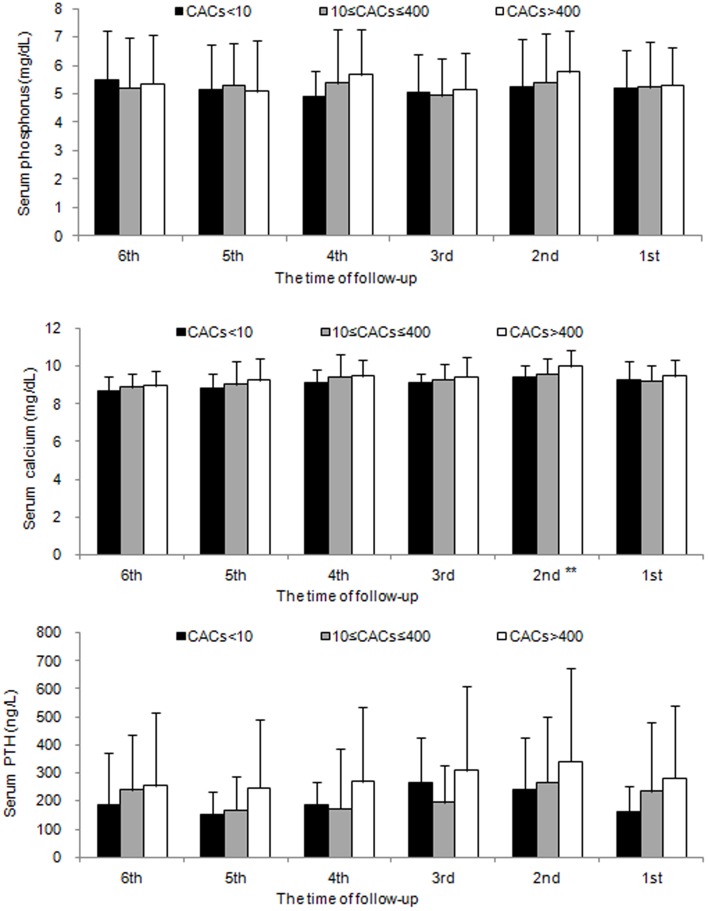
Effect of serum levels of phosphorus (top), calcium (middle), and parathyroid hormone (PTH, bottom) on CAC score during the past 6 follow-ups. The only significant differences were in the 2^nd^ measurement for serum calcium (^**^ *P*<0.05). Each column represents mean±SD. 1^st^ : the value at the entry of study (July, 2010); 2^nd^: the value of April, 2010; 3^rd^ : the value of January, 2010; 4^th^ : the value of October 2009; 5^th^ : the value of July 2009; 6^th^ : the value of April 2009.

To exclude the possibility that these results may be due to occasional or spurious events, we compared the serum levels of phosphorus, calcium, and PTH of the past 6 follow-ups (including the value at entry) in these three groups (Figure 2). Similarly, there were no significant differences among the three CAC groups in these parameters except for the second follow-up value for calcium.

### Variation in serum phosphorus, calcium, and PTH and severity of CAC

**Figure 3:**
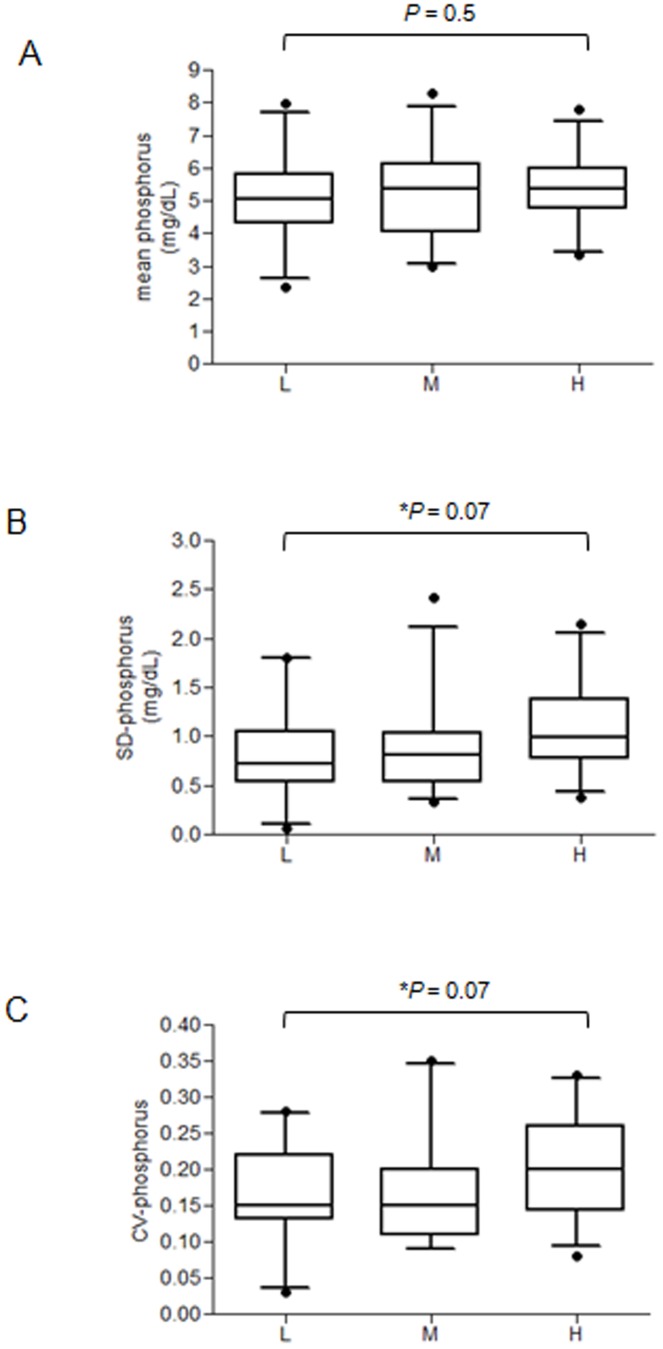
Boxplots of mean serum phosphorus (A), SD of serum phosphorus (B), and CV of serum phosphorus (C) in the three groups. The mean, SD, and CV were calculated from the past 6 follow-up values (April 2009 to July 2010). Data were compared by univariate ordinal logistic regressions. ^*^ *P*<0.2. Box: interquartile range; upper and lower lines: 75^th^ and 25^th^ percentiles; central horizontal line: median; vertical whiskers above and below the boxes: 5^th^ and 95^th^ percentiles; circles beyond the whiskers: outliers.

Table 3 shows that the mean value of serum phosphorus from the past 6 follow-ups (April 2009 to July 2010) was unrelated to CAC severity. The variability for serum phosphorus, defined by the SD and CV, tended to be greater in high-CACs group, but this was not significant (*p* = 0.07 for SD, *p* = 0.07 for CV). Similarly, the severity of CAC tended to be greater in patients with higher mean of calcium and PTH, but these differences were not significant (*p* = 0.2 for mean of calcium, *p* = 0.2 for mean of PTH). There were no significant differences among groups in the variabilities of calcium and PTH. We also performed boxplot analysis of this data (Figure 3).

**Table 3:** Mean, standard deviation (SD), and coefficient of variation (CV) of serum phosphorus, calcium, and iPTH of MHD patients by CAC score tertile.

	CAC score			
	<10 (n = 23)	11–400 (n = 26)	>400 (n = 28)	*P*
**Serum phosphorus**				
mean (mg/dL)	5.14±1.17	5.24±1.35	5.37±1.12	0.5
SD (mg/dL)	0.85±0.44	0.88±0.46	1.09±0.43	0.07*
CV	0.16±0.07	0.17±0.08	0.20±0.07	0.07*
**Serum calcium**				
mean (mg/dL)	9.14±0.51	9.24±0.76	9.44±0.74	0.2*
SD (mg/dL)	0.64±0.25	0.64±0.33	0.63±0.33	0.8
CV	0.07±0.03	0.07±0.04	0.07±0.03	0.7
**Serum PTH**				
mean (pg/mL)	205.3±99.2	207.1±154.8	281.5±253.5	0.2*
SD (pg/mL)	97.5±73.0	90.8±92.8	103.3±69.8	0.8
CV	0.47±0.26	0.45±0.28	0.44±0.27	0.8

Values expressed as *mean ± SD*. SD and CV were calculated from the past 6 follow-up values (April 2009 to July 2010). The *mean* was the average of patients' SD or CV which depended on the variations of patients over time. The *SD* showed how much variation from the average existed in one group.

MHD, maintenance hemodialysis; CAC, coronary artery calcification; SD, standard deviation; CV, coefficient of variation; PTH, parathyroid hormone.

Data compared by univariate ordinal logistic regressions;

*P<0.2.

### Independent predictors of CAC

To avoid model overfitting and the possible effects of confounding factors, all variables with *p*-values less than 0.2 under the univariate analysis (asterisks in Table 1, Table 2, and Table 3) were considered as potential predictors in the multivariate analysis (Table 4). In view of the strong positive correlation between phosphorus SD and CV (r = 0.85; *p*<0.001), we entered these two variables into separate regression models (Model 1 and 2, respectively). The results of the multivariate analysis indicated that female gend\er (Model 1, *p* = 0.002; Model 2, *p* = 0.003), age (Model 1, *p* = 0.003; Model 2, *p* = 0.01), serum FGF23 (Model 1, *p* = 0.003; Model 2, *p*<0.001), SD-phosphorus (Model 1, *p* = 0.007), and CV-phosphorus (Model 2, *p* = 0.01) were significantly and independently associated with CAC. Female gender was associated with a nearly 80% relative risk reduction (OR_Model 1_ = 0.20, OR_Model 2_ = 0.21). In Model 1, an increase of 1 year in age, 1 pg/mL in FGF23, or 1 mg/dL in the SD of serum phosphorus was associated with a 2.31, 2.25, and 2.12 increased risk of being in higher CAC category, respectively.

**Table 4:** Multiple logistic regression analysis of factors associated with CAC score tertile in MHD patients.

Variable	Unadjusted		Adjusted			
			Model 1		Model 2	
	OR	*P*	OR_1_ (95% CI)	*P_1_*	OR_2_ (95% CI)	*P_2_*
Female gender	0.28	<0.001	0.20 (0.07–0.55)	0.002	0.21 (0.07–0.58)	0.003
Age (/1-y)	1.82	0.01	2.31 (1.32–4.04)	0.003	2.04 (1.18–3.53)	0.01
Smoking	2.76	0.05				
**Laboratory measurements**						
Ferritin (ng/ml)	1.002	0.1				
Alb (/1-g/dL)	0.60	0.02				
CRP (/1-mg/L)	2.40	0.04				
PTH (/1-pg/mL)	1.53	0.09				
FGF23 (/1-pg/mL)	1.45	0.1	2.25 (1.31–3.85)	0.003	2.50 (1.46–4.30)	<0.001
HDL (/1-mg/dL)	0.57	0.03				
**Mean and variability measurements**						
Mean-PTH (/1-pg/mL)	1.43	0.2				
Mean-Ca (/1-mg/dL)	1.38	0.2				
SD-Pi (/1-mg/dL)	1.54	0.07	2.12 (1.23–3.63)	0.007		
CV-Pi (/1)	1.59	0.07			1.90 (1.16–3.11)	0.01

SD and CV were calculated from the past 6 follow-up values.

MHD, maintenance hemodialysis; CAC, coronary artery calcification; OR, Odds ratio; CI, confidence interval; Alb, albumin; CRP, C-reactive protein; PTH, parathyroid hormone; FGF23, fibroblast growth factor 23; HDL, high-density lipoprotein; SD, standard deviation; CV, coefficient of variation.

*P_1_* from multiple regression that adjusted for female sex, age, FGF23, and SD-phosphate.

*P_2_* from multiple regression that adjusted for female sex, age, FGF23, and CV-phosphate.

Finally, we analyzed whether mean and variability of serum phosphorus calculated from different follow-up durations affected the results of this multivariate analysis. Thus, we analyzed mean and variability values calculated from past 7 (January 2009 to July 2010), 8 (October 2008 to July 2010), 9 (July 2008 to July 2010), 10 (April 2008 to July 2010), and 11 (January 2008 to July 2010) follow-up values, and developed five other logistic regression models using the same procedures as described in Table 4. The results of multivariate analysis confirmed that age, sex, serum FGF23, and variability of serum phosphorus were independent predictors of CAC in HD patients (Table 5 and Table 6). Interestingly, there were no obvious differences in the ORs of phosphorus variabilities in all six models. This suggests that phosphorus variability calculated from the past 6 records alone might be sufficient.

**Table 5:** Odds ratio (OR) for risk of high CAC score in MHD patients.

Variable	Adjusted									
	SD was calculated from the past									
	7 records		8 records		9 records		10 records		11 records	
	OR	*P_1_*	OR	*P_1_*	OR	*P_1_*	OR	*P_1_*	OR	*P_1_*
Female gender	0.19	0.002	0.18	0.002	0.19	0.002	0.20	0.003	0.20	0.003
Age (/1-y)	2.26	0.005	2.20	0.006	2.22	0.006	2.25	0.006	2.43	0.003
FGF23 (/1-pg/mL)	2.31	0.003	2.29	0.004	2.29	0.004	2.25	0.005	2.25	0.005
SD-Pi (/1-mg/dL)	2.41	0.003	2.34	0.004	2.18	0.006	2.15	0.007	2.09	0.009

Variability in serum phosphorus was defined as the standard deviation.

MHD, maintenance hemodialysis; CAC, coronary artery calcification; OR, Odds ratio; FGF23, fibroblast growth factor 23; SD, standard deviation.

*P_1_* from multiple regression that adjusted for female sex, age, FGF23, and SD-phosphate.

**Table 6:** Odds ratio (OR) for risk of high CAC score in MHD patients.

Variable	Adjusted									
	CV was calculated from the past									
	7 records		8 records		9 records		10 records		11 records	
	OR	*P_2_*	OR	*P_2_*	OR	*P_2_*	OR	*P_2_*	OR	*P_2_*
Female gender	0.20	0.003	0.19	0.002	0.20	0.003	0.21	0.003	0.20	0.003
Age (/1-y)	1.97	0.02	1.91	0.02	1.95	0.02	2.03	0.01	2.22	0.007
FGF23 (/1-pg/mL)	2.85	<0.001	2.79	<0.001	2.76	<0.001	2.68	<0.001	2.73	<0.001
CV-Pi (/1)	2.32	0.002	2.25	0.002	2.20	0.003	2.18	0.003	2.12	0.003

Variability in serum phosphorus was defined as the coefficient of variation.

MHD, maintenance hemodialysis; CAC, coronary artery calcification; OR, Odds ratio; FGF23, fibroblast growth factor 23; CV, coefficient of variation.

*P_2_* from multiple regression that adjusted for female sex, age, FGF23, and CV-phosphate.

## Discussion

Dialysis patients have a 10- to 20-fold increased mortality in comparison with healthy individuals, cardiomyopathy is one of the most important causes of this increased mortality [35]. The association between vascular calcification and left ventricular hypertrophy (LVH) had also been reported in many studies, among which the CAC was considered as one of risk factors of promoting the development of LVH [36], [37]. CAC occurs more frequently in HD patients than in non-HD subjects of the same age and sex. In CKD patients, CAC can be measured by many ways, among which CT-based CAC score is recommend as a “standard” detection by ISN KDIGO guideline [38]. The Multi-Ethnic Study of Atherosclerosis (MESA) study reported that the prevalence of coronary calcification (Agatston score>0) was 59% in Chinese men and 42% in Chinese women who were 45 to 84 years-old and had no clinical cardiovascular disease [39]. By comparison, other studies reported the prevalence of CAC in HD patients was 72 to 83% [1]–[3], and that there was a higher prevalence (88%) in young HD patients (20 to 30 years-old) [13]. Our results indicated that 78% of MHD patients had CACs more than 0, supporting the high prevalence of coronary calcification in this population. CAC can be associated with severe clinical consequences, and maybe considered a predictor of CVD [4]–[7], which could account for approximately half of all deaths among HD patients [8], [9]. Previous cross-sectional and prospective clinical studies have investigated the factors that contributed to CAC, such as the classic cardiovascular risk factors and factors related to uremia and the HD treatment itself [1]–[3], [11]–[17]. The present study is the first to report a significant and independent association between variation of serum phosphorus and CAC. These results may help to identify the presence of modifiable risk factors of HD patients in clinic practice.

In agreement with previous research, we observed that age [3], [11], [21] and gender [7], [39]were the most important risk factors for coronary calcification [40]. Age-linked vascular calcification has been known since the nineteenth century. In particular, CAC appears in the artery wall but not in other soft tissues, as serum calcium and phosphorus levels increase with age [41]. On the other hand, age is also a reflection of the cumulative exposure to all atherogenic risk factors for calcification [42]. Among female hormones, oestrogen has known beneficial effects on lipid and bone metabolism which may delay the process of calcification [43], [44].

Patients with diabetes mellitus have a greater prevalence of hyperglycemia, oxidative stress, insulin resistance, and inflammation, and these may play a role in vascular calcification [45]–[48]. We observed no significant association between diabetes and coronary calcification, although only 11 of our 77 patients had diabetes and the proportion of diabetes patients was higher in the high CACs group. Other studies have also reported no relationship of diabetes and calcification [15], [49]. The association between these factors may be clarified by measurement of the serum levels of glucose or metabolism-associated proteins that are responsible for vascular calcification in the presence of diabetes [45]–[48].

As in some previous studies, we also found no significant associations between dialysis vintage, blood pressure, Kt/V, BMI, acidosis, serum creatinine, blood urea nitrogen, or hemoglobin with coronary calcification [2], [13], [50], [51]. This may be because some of the parameters were strictly controlled in a relatively narrow range among subjects in the current study. Previous research indicated that smoking, medications, iron metabolism, nutritional state, inflammation, and dyslipidemia impact the severity of coronary calcification in HD patients [18], [52]–[55]. However, our multivariate analysis showed that GFR, serum levels of ferritin, transferrin saturation, albumin, CRP, HDL, and medications were not significantly associated with CAC, possibly because of the limited cohort size in this study. Though the smoking showed a marginal trend toward significance (*p* = 0.05) in univariate analysis, it was excluded from the final model possibly because the difference of smoking among the three groups was due to the relationship between smoking and gender, not real smoking and CAC.

At present, mineral disturbances, including hyperphosphatemia and hypercalcaemia, secondary hyperparathyroidism, high FGF23, and adynamic bone disease, are the best known and studied uremic abnormalities associated with development of vascular calcifications. Experimental studies indicated that elevated calcium and phosphorus had direct effects on vascular smooth muscle cells (VSMCs) that promote vascular calcification, including stimulation of osteogenic/chondrogenic differentiation, vesicle release, apoptosis, loss of inhibitors, and extracellular matrix degradation [24]. Indeed, some (but not all) clinical studies of HD patients indicated that serum calcium levels were associated with vascular calcification [1], [2], [11], [15], [21], [22]. Moreover, a recent meta-analysis showed that serum phosphorus – but not calcium or PTH – was associated with cardiovascular events and mortality in individuals with CKD (N = 327 644) [56]. In agreement with these results, we also found no significant association between serum calcium and CACs. The role of serum calcium in artery calcification of HD patients requires further study.

Some epidemiological evidence suggests a pivotal role for elevated serum phosphorus in driving vascular calcification in ESRD patients [2], [13], [57], [58]. For example, a cross-sectional analysis by Raggi *et al.* [2] indicated that serum phosphorus was significantly and positively correlated with the severity of CAC in adult HD patients. Other studies indicated that this relationship also occurs in pediatric and young HD patients [13], [57], [58]. However, some other studies reported no significant association between serum phosphorus and CAC in HD patients [1], [11], [15], [21], [22]. Our results also indicated no significant association between CAC and hyperphosphatemia.

One possible reason for the disagreement about the role of serum phosphorus in vascular calcification may be that the HD patients of these different studies had different baseline levels of serum phosphorus and calcium. For example, the mean values of serum phosphorus and the calcium–phosphorus product of all patients in the present study were 5.2 mg/dL (±1.4) and 48.9 mg^2^/dL^2^ (±14.1), respectively; the corresponding values in the Goodman *et al.* [13] study were 6.9 mg/dL (±0.9) and 65.0 mg^2^/dL^2^ (±10.6) and the values in the Raggi *et al.* [2] study were 5.7 mg/dL (±1.4) and 55.1 mg^2^/dL^2^ (±13.5). The higher levels of serum phosphorus reported in these two studies may have stimulated phenotypic changes, an osteoblastic transcriptional program, or apoptosis of smooth muscle cells of the vascular system [59]–[61].

Another possible reason for the disagreement about the role of serum phosphorus in vascular calcification may be that a single measurement or mean value of serum phosphorus does not indicate changes in phosphorus metabolism over time. Our results clearly showed that the variability of serum phosphorus was positively associated with coronary calcification in HD patients, even though the mean serum phosphorus levels were not very high. This suggests that phosphorus variability may better reflect the imbalance of phosphorus metabolism in HD patients. Phosphorus regulates enzymatic activity and serves as an essential component of nucleic acids, adenosine triphosphate, and phospholipid membranes [62], and phosphorus variability may induce cell apoptosis, a key regulator of VSMC calcification [63]. In addition, high variability of serum phosphorus might be associated with low rate of bone turnover, because bone serves as a huge phosphorus reservoir that contributes to serum phosphorus stability *via* bone formation and resorption [24], [64]. Under the state of low bone turnover, excess phosphorus cannot be taken up by the adynamic bone, so it might precipitate on vascular tissue, or move into a newly formed exchangeable phosphorus pool that develops as VSMCs transition towards the osteoblastic phenotype during medial calcification in vascular tissues of CKD patients [65]. Preliminary data indicate a negative relationship between low bone turnover and vascular calcification in HD patients [66]. More studies are needed to establish whether phosphorus variability is simply a marker of CAC or whether phosphorus variability is part of the mechanism for vascular calcification in HD patients.

There is disagreement about relationship between serum PTH and vascular calcification. Some previous studies showed that low PTH (median 200 pg/mL) or higher PTH (>300 pg/mL) was associated with increased risk of vascular calcification in HD patients [49], [66]–[69], but whether caused by the related bone disease was difficult to clarify. In addition, some (but not all) studies reported reduction in the rate of progression of vascular calcification after parathyroidectomy [70]. The present study found no association of coronary calcification with serum PTH (mean value in three groups ranged from 150 to 350 pg/mL), possibly because the predictive value of PTH for identifying underlying bone histology had getting weaker [71], and it was difficult to judge whether high or low PTH level was the consequence or cause of calcium salt use, vitamin D analogue use, parathyroidectomy, and hyperphosphataemia, all of which can affect vascular calcification [70].

Our results demonstrated that serum FGF23 was significantly related to the severity of CAC in HD patients, in accordance with previous cross-sectional studies [16], [57], [68], [72]–[75]. There is no clear evidence indicating a direct pathogenetic effect of FGF23 on vasculature, so previous studies attributed this association to indirect effects. In particular, elevated FGF23 may reflect a higher time-averaged phosphorus burden, vitamin D deficiency, different dose of phosphorus binders [72], lower adiponectin, and dyslipidemia [74]. Recent mechanistic studies indicated that Klotho, a β-glucuronidase that affects calcium homeostasis, was an inhibitor of vascular calcification [76], [77]. We observed no differences in phosphorus and vitamin D in our three CACs groups, so we suggest that circulating FGF23 may have a protective effect on arterial wall integrity and that rising FGF23 levels in HD patients are in part a consequence of vascular resistance to FGF23, because of uremia-mediated down-regulation of Klotho expression in vascular cells [77], [78]. Future studies are needed to examine the existence of FGF-23/Klotho interactions within the arterial wall in order to more completely characterize the anticalcific effects of FGF23.

We acknowledge several limitations of the present study. First, we cannot infer the causality of the associations identified in current analysis due to the possible presence of unknown confounders. Second, the sample size was rather small and all patients were from a single institution, so there may have been some selection bias. Third, the variability of phosphorus calculated from trimonthly records may not be the most accurate way to assess phosphorus fluctuation. Fourth, we did not measure the level of bone-associated proteins, such as members of the BMP family (BMP-2, BMP-7), which are known to be involved in bone metabolism and vascular calcification [52], [79], so we were unable to determine whether phosphorus variability was a marker of bone metabolism or direct affected vasculature. Fifth, the quantification of CAC has only been performed by a single reader, the biased reading may have happened. Finally, we have no data on alcohol consumption which may be a risk factor of vascular calcification [36], [80].

In conclusion, variability of serum phosphorus appears to contribute significantly to CAC in MHD patients. Future clinical studies are needed to establish whether maintenance of a stable serum phosphorus level decreases coronary calcification and associated morbidity and mortality in MHD patients.
